# ncStem: a comprehensive resource of curated and predicted ncRNAs in cancer stemness

**DOI:** 10.1093/database/baae081

**Published:** 2024-08-13

**Authors:** Hui Liu, Nan Zhang, Yijie Jia, Jun Wang, Aokun Ye, Siru Yang, Honghan Zhou, Yingli Lv, Chaohan Xu, Shuyuan Wang

**Affiliations:** College of Bioinformatics Science and Technology, Harbin Medical University, Harbin, Heilongjiang 150081, China; College of Bioinformatics Science and Technology, Harbin Medical University, Harbin, Heilongjiang 150081, China; College of Bioinformatics Science and Technology, Harbin Medical University, Harbin, Heilongjiang 150081, China; College of Bioinformatics Science and Technology, Harbin Medical University, Harbin, Heilongjiang 150081, China; College of Bioinformatics Science and Technology, Harbin Medical University, Harbin, Heilongjiang 150081, China; College of Bioinformatics Science and Technology, Harbin Medical University, Harbin, Heilongjiang 150081, China; College of Bioinformatics Science and Technology, Harbin Medical University, Harbin, Heilongjiang 150081, China; College of Bioinformatics Science and Technology, Harbin Medical University, Harbin, Heilongjiang 150081, China; College of Bioinformatics Science and Technology, Harbin Medical University, Harbin, Heilongjiang 150081, China; College of Bioinformatics Science and Technology, Harbin Medical University, Harbin, Heilongjiang 150081, China

## Abstract

Cancer stemness plays an important role in cancer initiation and progression, and is the major cause of tumor invasion, metastasis, recurrence, and poor prognosis. Non-coding RNAs (ncRNAs) are a class of RNA transcripts that generally cannot encode proteins and have been demonstrated to play a critical role in regulating cancer stemness. Here, we developed the ncStem database to record manually curated and predicted ncRNAs associated with cancer stemness. In total, ncStem contains 645 experimentally verified entries, including 159 long non-coding RNAs (lncRNAs), 254 microRNAs (miRNAs), 39 circular RNAs (circRNAs), and 5 other ncRNAs. The detailed information of each entry includes the ncRNA name, ncRNA identifier, disease, reference, expression direction, tissue, species, and so on. In addition, ncStem also provides computationally predicted cancer stemness-associated ncRNAs for 33 TCGA cancers, which were prioritized using the random walk with restart (RWR) algorithm based on regulatory and co-expression networks. The total predicted cancer stemness-associated ncRNAs included 11 132 lncRNAs and 972 miRNAs. Moreover, ncStem provides tools for functional enrichment analysis, survival analysis, and cell location interrogation for cancer stemness-associated ncRNAs. In summary, ncStem provides a platform to retrieve cancer stemness-associated ncRNAs, which may facilitate research on cancer stemness and offer potential targets for cancer treatment.

**Database URL**: http://www.nidmarker-db.cn/ncStem/index.html.

## Introduction

The existence of cancer stem cells (CSCs) endows the tumor with stem-like properties, known as cancer stemness, arising a paradigm shift in the understanding of cancer biology and therapy. Cancer stemness is thought to be responsible for tumor initiation, metastasis, heterogeneity, recurrence, and resistance to standard cancer therapies [1–3]. Numerous intrinsic and extrinsic factors have been identified to modulate cancer stemness, including genetic and epigenetic factors, signaling pathways, tumor microenvironment, and so on [4, 5]. It has been suggested that targeting CSCs may be critical to achieve long-term remission and prevent cancer recurrence [2]. Understanding the molecular and cellular mechanisms underlying cancer stemness will contribute to a deeper understanding of cancer development and progression and facilitate the development of targeted therapy for cancer.

Non-coding RNAs (ncRNAs) are a diverse class of RNA molecules that are generally not capable of coding for proteins, containing microRNAs (miRNAs), long non-coding RNAs (lncRNAs), and circular RNAs (circRNAs). ncRNAs are increasingly recognized as key regulators of various diseases and biological processes, including involvement in cancer stemness [6–8]. A growing number of studies have identified numerous ncRNAs involved in cancer stemness. However, the scattered information has made it inconvenient for researchers to comprehensively characterize the ncRNAs associated with cancer stemness.

**Figure 1.**
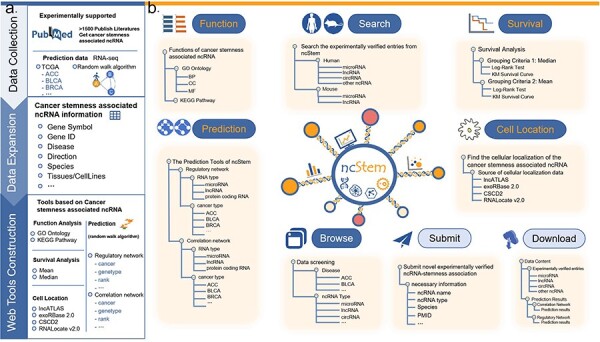
Database content and user interface of ncStem. (a) Summary of the contents of the database, including the collection of articles reporting ncRNAs in cancer stemness, prediction of cancer stemness-associated ncRNAs, and the construction of online web tools. (b) User interface of ncStem, which provides flexible ways to browse, search, analyze, and download all curated and predicted ncRNAs in cancer stemness.

To fill this gap, we have developed the ncRNA in Cancer Stemness database (ncStem, http://www.nidmarker-db.cn/ncStem/). ncStem manually curates experimentally verified ncRNAs involved in cancer stemness. In addition, computationally predicted cancer stemness-associated ncRNAs are also provided in ncStem. Furthermore, functional enrichment analysis, survival analysis, and cellular location display tools are also integrated in ncStem (Fig. 1). Overall, ncStem offers a web server for browsing and analyzing ncRNAs in cancer stemness, which might facilitate the cancer stemness research and discovery of potential targets for cancer treatment.

## Data collection and processing

### Experimentally verified data collection

To collect experimentally verified ncRNAs associated with cancer stemness, we queried publications on ncRNAs and cancer stemness from the PubMed database using a list of keywords, including “cancer stemness and microRNA” or “cancer stem cell and microRNA” or “cancer stemness and circular RNA” or “cancer stem cell and circular RNA” or “cancer stemness and lncRNA” or “cancer stem cell and lncRNA” or “cancer stemness and non-coding RNA” or “cancer stem cell and non-coding RNA.” More than 1500 articles were downloaded from the PubMed database (before December 2023). Each publication was then carefully reviewed. After filtering out the irrelevant articles, ncStem collected 645 entries on cancer stemness-associated ncRNAs, including 159 lncRNAs, 254 miRNAs, 39 circRNAs, 4 small nucleolar RNAs, and 1 piwi-interacting RNA in 2 species (human and mouse). The detailed information of each entry contains the ncRNA name, ncRNA identifier, reference, species, disease, tissue/cell line, direction, description, PubMed identifier, and so on.

### Prediction of cancer stemness-associated ncRNAs

To expand the potential ncRNAs involved in cancer stemness, we proposed “Prediction Tools” to predict cancer stemness-associated ncRNAs. Many studies have shown that network-based methods are reliable in establishing biological associations [9–11]. Therefore, we constructed cancer-specific co-expression and regulatory networks, and used the random walk with restart (RWR) algorithm to prioritize the ncRNAs-associated with cancer stemness. We first recruited 26 stemness protein-coding gene (PCG) sets from StemChecker [12], a database with the most comprehensive and up-to-date collection of published stemness signatures. Then the PCGs that appeared in at least three datasets were considered as stemness-associated genes, and all over 727 PCGs were obtained. All these obtained PCGs, combined with our curated human cancer stemness-associated ncRNAs, were used as seeds for the RWR algorithm (Supplementary Table S1). The cancer-specific co-expression network, comprising PCG–PCG, PCG–lncRNA, miRNA–PCG, and miRNA–lncRNA pairs, was constructed by co-expression pairs calculated from the transcriptional profiles of the 33 Cancer Genome Atlas (TCGA) cancer types. For each TCGA cancer type, the genes (including PCGs, lncRNAs, and miRNAs) that belonged to the bottom 25% of expression variances were deleted. The remaining genes were paired and the Pearson correlation coefficient (PCC) values were calculated and ranked respectively. The PCG–PCG and PCG–lncRNA pairs were ranked from highest to lowest based on absolute PCC values, while miRNA–PCG and miRNA–lncRNA pairs were ranked from negative to positive based on PCC values. The top ranked pairs were reserved for the co-expression networks. To get a flexible and comparable prediction results, different cutoff values were adopted and the top ranked 0.1%, 1%, 5%, 10%, and 20% pairs were reserved as co-expression pairs. We ultimately retained the pairs that contained at least one seed gene to construct the final cancer-specific co-expression networks. The cancer-specific regulatory network for 33 TCGA cancer types was constructed following our previously published work, comprising eight types of regulatory relationships among four types of factors [transcription factors (TFs), miRNAs, lncRNAs, and PCGs] [13]. The eight types of regulatory interactions, including TF–miRNA, TF–lncRNA, TF–PCG, miRNA–lncRNA, miRNA–TF, miRNA–PCG, lncRNA–TF, and lncRNA–PCG, were downloaded and integrated from corresponding credibly curated databases, such as TransmiR (v2.0), ChIPBase, miRecords, miRTarBase, LncBase (v2.0), LncRNA2Target (v2.0), and so on. The obtained regulatory interactions were intersected with co-expression pairs in each cancer type to construct the final cancer-specific regulatory network. We also provided five cutoff values (0.1%, 1%, 5%, 10%, and 20%) for the regulatory networks. We denoted *W* as the probability transition matrix, and it was derived from the adjacency matrix of corresponding network. The formula was defined as:


$$w(i,j) = \left\{ {\begin{array}{*{20}{c}}
{A(i,j)/\sum\nolimits_j {A(i,j),} }&{if\;\sum\nolimits_j {A(i,j) \ne 0} }\\
{0,}&{otherwise}
\end{array}} \right.$$

where *w* (*i, j*) represented the element in the probability transition matrix, and *A* (*i, j*) represented the element in the adjacency matrix. Let *P*_0_ denote the initial probability vector and *P_t_* denote the probability vector in step *t*. Let *α* be the restart probability of the random walk in each step. The formula of the random walk with restart algorithm can be defined as follows:


$${p_{t + 1}} = (1 - \alpha )W{p_t} + \alpha {p_0}$$

Here, the *α* was set to 0.5, and the initial probability of each seed node was set to 1/n (where n was the number of total seed factors) while all other nodes in the network were set to 0. The final stable probabilities were used to measure the proximity of each node to the seed nodes. In order to select significant prediction results, we randomly select pseudo seeds with the same number as the real seeds, and then calculated random walk probabilities for other nodes. Repeated this process 1000 times, and considered the predicted genes whose true probability was in the top 5% of all scores as significant cancer stemness-associated genes. We reviewed the scale of the networks at different thresholds and the corresponding prediction results. As expected, the size of the network expanded as the threshold value increased, and the number of predicted ncRNAs also increased with the expansion of network size (Supplementary Table S2). We also examined the overlap of prediction results at different thresholds, and found that most of the ncRNAs predicted by smaller networks were also included in the prediction results of larger networks (Supplementary Figure S1). In addition, we obtained the differentially expressed ncRNAs (DEncRNAs_LHSI) between TCGA cancer samples with low stemness index and high stemness index for each cancer type (with the median as the threshold, |log2FC| > 1 and False Discovery Rate (FDR) < 0.05). The stemness indices of TCGA cancer samples were calculated using the mRNAsi proposed by Malta *et al*. [14], which perform a one-class logistic regression on the mRNA expression data and ranged from 0 to 1, with a value closer to 1 indicating stronger stem cell characteristics. The cancer stemness-associated ncRNAs predicted by co-expression networks in all cancers and regulatory networks in most cancers (19/33) were significantly enriched in DEncRNAs_LHSI (Supplementary Table S3). We further retrieved differentially expressed ncRNAs (DEncRNAs_TN) between tumor and normal samples for each cancer type from GEPIA2 (|log_2_FC|> 1, q-value < 0.01) [15], an enhanced web server that performs a combined analysis of the TCGA and GTEx datasets. The cancer stemness-associated ncRNAs predicted by co-expression networks for each cancer type were all significantly enriched in DEncRNAs_TN (Supplementary Table S3). Although the results predicted by the regulatory networks were not significantly enriched, recently published literature has demonstrated that several of the top ranked ncRNAs are closely associated with tumor initiation, progression, and metastasis. For example, hsa-let-7b-5p is predicted to be ranked first in breast invasive carcinoma (BRCA) by the 0.1% BRCA regulatory network and Shao *et al*. have shown that AC074117.1/hsa-let-7b-5p axis-mediated high expression of SLC35A2 acts as a tumor promoter in BRCA via extracellular signal regulated kinase signaling, promoting BRCA progression [16]. Hsa-miR-26a-5p is predicted to be ranked first in lung adenocarcinoma (LUAD) by the 0.1% LUAD regulatory network and Son *et al*. have shown that over-expression of miR-26a-5p leads to a marked reduction of colony formation and sphere formation in lung cancer, suggesting that miR-26a-5p can suppress cancer stem cell formation in lung cancer [17]. Therefore, we can cautiously conclude that the RWR algorithm can reasonably optimize cancer stemness-associated ncRNAs. In total, we obtained 12 104 predicted entries, including 11 132 lncRNAs and 972 miRNAs across 33 TCGA cancers. All these prioritized entries were provided in the ncStem database and users can retrieve the predicted cancer stemness-associated ncRNAs by the customized criteria.

### Database construction and user interface

ncStem carried out the data management with the software MySQL (v8.0.30). The web pages were constructed with Java Server Pages and deployed on the Apache Tomcat web server (v9). Several Java Script plugins such as jQuery (v2.2.3), Datatable (1.10.10), ECharts (v4.0), and Plotly (v2.20.0) were used for the creation and visualization of data tables. All statistical analyses were performed with the R framework (v4.3.2).

**Figure 2.**
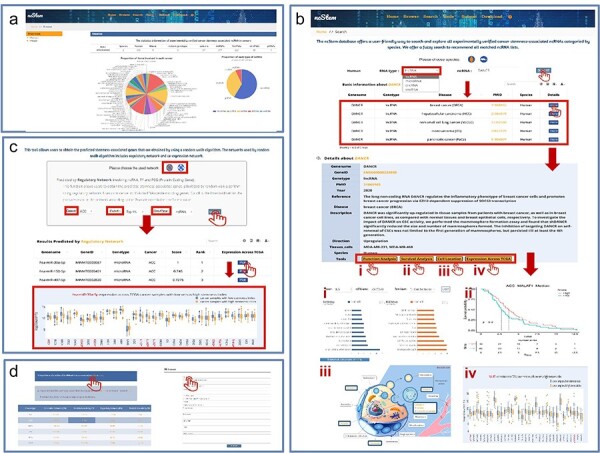
A comprehensive view of ncStem database. (a) Interface of the “Browse” page. Users can comprehensively browse all experimentally validated cancer stemness-associated ncRNAs, categorized by ncRNA type, and disease across two species. (b) Interface of the “Search” page. Users can search all experimentally validated entries by selecting the RNA type and species of interest. The detailed information page provides “Function Analysis,” “Survival Analysis,” “Cell Location,” and “Expression across TCGA” tools for further investigation of the queried ncRNA. (c) Interface of the tool “Prediction” page. Users can achieve the results predicted by the two networks by selecting either “Regulatory Network” or “Co-expression Network.” (d) Download and Submit page. All of the experimentally verified entries and predicted entries can be downloaded freely. In addition, users can submit novel stemness-associated ncRNAs to ncStem.

The ncStem offers an interface to freely browse, search, analyze and download all curated ncRNAs in cancer stemness. On the “Home” page, users can get a brief overview of the ncStem database and the statistics of the recorded ncRNAs. A “quick search” engine is available to retrieve data directly. A panel of online tools can be directly accessed on the “Home” page, including “Prediction,” “Function,” “Survival,” and “Cell Location,” which are also integrated into the “Tools” navigation. On the “BROWSE” page, all experimentally validated cancer stemness-associated ncRNAs can be comprehensively viewed, categorized by ncRNA type and disease across two species (Fig. 2a). On the “Search” page, users can search all experimentally validated entries by selecting the RNA type and species of interest, and inputting the queried ncRNA. We provide a fuzzy search to recommend all matching ncRNA listings (Fig. 2b). On the result page, the matching entries are displayed by cancer type. Users can click “more” to query detailed information about the corresponding entry. The tools “Function,” “Survival,” and “Cell Location” for further investigation of the queried ncRNA can be accessed directly on the detail page (Fig. 2b I, II, III). The “Function” tool employed gene set enrichment analysis method to perform functional enrichment analysis for the queried ncRNA based on the gene list ranked by PCC values across 33 TCGA cancer types, using R package clusterProfiler. Top enriched Gene Ontology and Kyoto Encyclopedia of Genes and Genomes terms can be viewed on the result page. The “Survival” tool performs online survival analysis and generates Kaplan–Meier survival curves for the queried ncRNA across 33 TCGA cancers. It is reported that RNAs with different subcellular localization exert their roles in tumor stemness through different mechanisms [18], and that the mislocalization of RNA can promote the development and progression of cancer [19]. For example, nuclear lncRNAs are mainly associated with chromatin organization, transcription, and RNA processing, whereas cytoplasmic lncRNAs mainly influence mRNA stability by acting as competing endogenous RNAs or interacting with RNA-binding proteins [18]. To better understand ncRNAs in tumor stemness, ncStem provided the “Cell Location” tool to display all possible subcellular and extracellular vesicle locations of the queried ncRNA collected from public data sources, including exoRBase (v2.0) [20], CSCD (v2.0) [21], RNALocate (v2.0) [22], and LncATLAS [23]. In addition, the expression of the queried ncRNA across TCGA cancer samples with low versus high stemness index is also displayed (with the median as the threshold). Cancers with significant expression difference are highlighted with red stars (|log_2_FC| > 1 and FDR < 0.05) (Fig. 2b IV). On the “Tools Prediction” page, users can retrieve the predicted cancer stemness-related ncRNAs prioritized by two different networks at different thresholds. One is the prioritized results based on the regulatory network. The other is the results based on the co-expression network. Users can view the detailed prediction results by selecting the cancer type and the ncRNA type (Fig. 2c). The results will display the basic information, including Genename, Genetype, Cancer, Score (obtained by RWR), Rank, etc. Similarly, we also display the expression of the queried ncRNA across TCGA cancer samples with low versus high stemness index. On the “Download” page, users can download all experimentally validated and predicted ncRNAs associated with cancer stemness (Fig. 2d). We also provide regulatory networks and co-expression networks at different thresholds employed by RWR for prediction in each cancer type. Two formats are available for downloading the data (Text and Excel). In addition, ncStem encourages its users to submit novel experimentally supported ncRNA in cancer stemness (Fig. 2d).

## Discussion

Cancer stemness refers to the presence of a small population of cells within a tumor that exhibit stem cell-like properties and are thought to drive tumor initiation, progression, and treatment resistance. ncRNAs have been identified as critical players in the regulation of cancer stemness, contributing to the aggressive behavior and therapeutic resistance of cancers. Comprehensively analyzing the intricate regulatory roles of these ncRNAs in cancer stemness could help to decipher the origin of tumor stem cells and facilitate the development of targeted therapies that effectively disrupt the mechanisms of cancer stem cell self-renewal and survival, thereby improving the prognosis for cancer patients undergoing treatment. Therefore, we developed ncStem, a specialized database comprising experimentally validated and computationally predicted ncRNAs associated with cancer stemness. Functional enrichment analysis, survival analysis, and cellular location display tools are also integrated in ncStem. These properties not only offer comprehensive insights but also provide valuable therapeutic targets for future drug development.

We believe that ncStem will facilitate the cancer stemness research and contribute to the discovery of potential targets for cancer treatment. We should also point out that some data may be missing as it is difficult to capture all relevant information during manual curation. In the future, we will continuously curate the scientific literature on ncRNAs associated with cancer stemness, and expand our data curation to other species. In addition, we will improve our database with more practical tools, such as a collection of small molecules targeting cancer stem cells, and ensure regular updates and usefulness.

## Supplementary Material

baae081_Supp

## Data Availability

All data in ncStem are stored in the download section, and users can download it on demand.

## CRediT statement

Hui Liu: Conceptualization, Visualization, Methodology, Writing—original draft. Nan Zhang: Visualization, Software, Writing—original draft. Yijie Jia: Investigation, Validation. Jun Wang: Data curation. Aokun Ye: Data curation. Siru Yang: Investigation, Validation. Honghan Zhou: Data review. Yingli Lv: Data review. Chaohan Xu: Conceptualization, Project administration, Writing—review & editing. Shuyuan Wang: Conceptualization, Project administration, Writing—review & editing, Supervision. All authors have read and approved the final manuscript.
