# Supplementary figures and images for: ncStem: a comprehensive resource of curated and predicted ncRNAs in cancer stemness
Source: Database (Oxford). 2024 Aug 13;2024:baae081. doi: 10.1093/database/baae081 (PMC11321241; doi:10.1093/database/baae081)

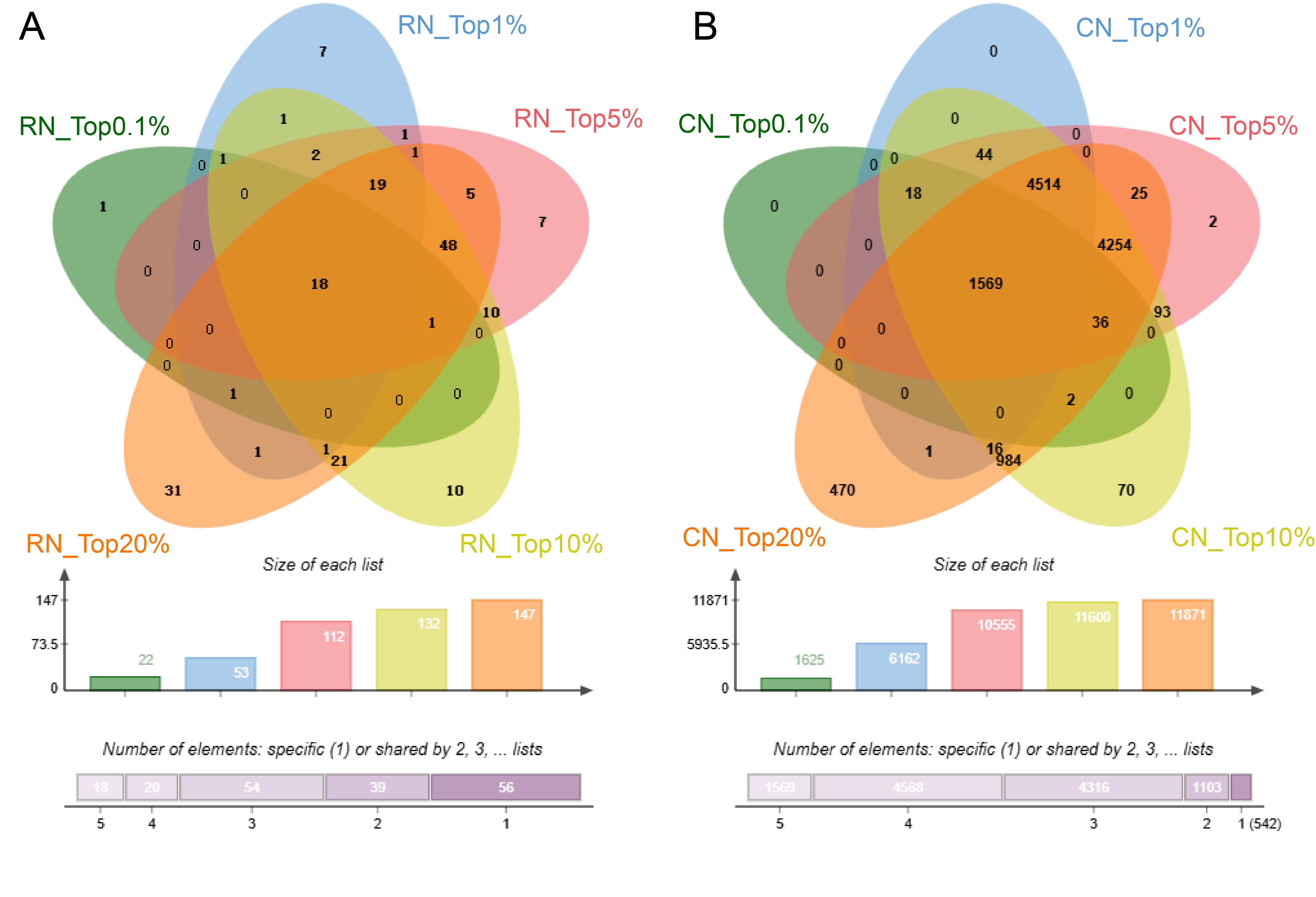

Supplement: baae081_Supp [file baae081_supp.zip › suppl_data/Supplementary_Figure_S1.jpeg]
